# Detection of Degraded Formulated BaTiO_3_ Induced by Oxygen Vacancy Migration Using Ultrasonic Attenuation

**DOI:** 10.1002/advs.77083

**Published:** 2026-08-14

**Authors:** Haley N. Jones, Clive A. Randall, Andrea P. Argüelles, Susan Trolier‐McKinstry

**Affiliations:** ^1^ Materials Science and Engineering The Pennsylvania State University University Park Pennsylvania USA; ^2^ Materials Research Institute The Pennsylvania State University University Park Pennsylvania USA; ^3^ Engineering Science and Mechanics The Pennsylvania State University University Park Pennsylvania USA

**Keywords:** attenuation, multilayer ceramic capacitors, oxygen vacancies, ultrasound

## Abstract

Insulation resistance degradation caused by electromigration of oxygen vacancies under DC bias is a common mechanism that limits lifetime in multilayer ceramic capacitors (MLCCs). This work explores the use of high frequency ultrasound (100 MHz) as a non‐electrical, nondestructive characterization tool to spatially identify regions of degradation caused by oxygen vacancy migration through ultrasonic attenuation, which results from acoustic impedance changes leading to scattering contrast. To complement this technique, thermally stimulated depolarization current (TSDC) measurements were utilized to both degrade parts and evaluate the presence of electromigrated oxygen vacancy space charge. Commercial 1812 BME X7R MLCCs (100 V rated, 560 nF) were characterized using a custom ultrasonic immersion setup in both a pristine state and after TSDC (degraded at 130°C, 140°C, or 150°C for 8 h and measured using a 10°C min^−1^ heating rate). Each TSDC condition induced oxygen vacancy migration across grain boundaries, which was confirmed using passive voltage contrast SEM. The average ultrasonic attenuation increased (1‐16.4%) after TSDC stress. Additionally, more extreme poling conditions resulted in larger variations in the spatial attenuation response. Thus, ultrasonic attenuation is sensitive to MLCC degradation caused by electromigration of oxygen vacancies and may be useful as a non‐electrical evaluation of this mechanism.

## Graphical abstract

Oxygen vacancy migration is a key degradation mechanism for the majority of MLCCs. There are limitations with spatially identifying regions of a capacitor that have degraded due to oxygen vacancy migration. This work showcases ultrasonic attenuation as a non‐electrical and nondestructive technique of identifying oxygen vacancy degraded regions of an MLCC.

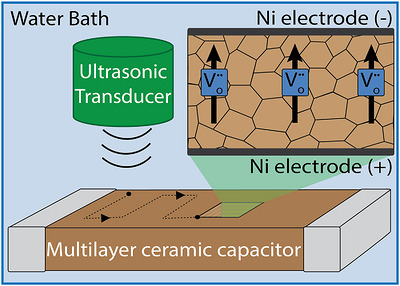

## 1 Introduction

There are many capacitors used in any electronic circuit; hence, capacitors often determine system reliability as they are responsible for approximately 30% of all electronic circuit failures [1]. One common degradation mechanism responsible for low lifetimes in class II capacitors is insulation resistance degradation (IR) caused by electromigration of oxygen vacancies under DC bias [2]. Formulated barium titanate (BaTiO_3_) is the dielectric material of choice for class II MLCCs due to its high and comparatively temperature‐stable dielectric permittivity [3, 4]. However, the long‐term reliability of BaTiO_3_‐based MLCCs is often compromised by oxygen vacancy migration [2, 4, 5]. Thus, understanding oxygen vacancy migration is crucial for improving the performance and durability of these capacitors.

Oxygen vacancies are intrinsic defects in BaTiO_3_ ceramics, typically introduced during the sintering process in reducing atmospheres, especially when base‐metal electrodes (BMEs) like nickel are used [5, 6]. A significant number of oxygen vacancies remain within the BaTiO_3_ despite subsequent re‐oxidation treatments; these vacancies can migrate toward the cathodes under an applied DC electric field, leading to various degradation phenomena. The electrical equivalent circuit of an MLCC with formulated BaTiO_3_ is often represented as a 4‐RC (resistor‐capacitor) circuit composed of the ceramic/internal electrode interfaces, grain boundaries, and core and shell regions of the core‐shell microstructure [6]. Insulation resistance degradation occurs as oxygen vacancies accumulate near the cathode side during aggressive degradation of MLCCs, which causes a Schottky barrier reduction at the cathode. Electrostatic charge balance of these oxygen vacancies then necessitates electron injection from the cathode. These electrons are typically trapped on the Ti ions, resulting in a change in the oxidation state of Ti ions from Ti^4+^ to Ti^3+^, and possibly Ti^3+^ to Ti^2+^; this in turn produces increased leakage current [4, 5, 7, 8, 9]. The assumed electrode reaction at the anode will inject additional oxygen vacancies ($V_{O}^{\cdot \cdot })$ according to the defect chemistry reaction:

(1) $$O_{O}=\frac{1}{2}\;O_{2}\left(g\right)+V_{O}^{\cdot \cdot }+2e^{\prime }\;$$

Decrease of IR over time can result in a short circuit of the MLCC and possible circuit failure; thus, understanding the underlying mechanisms of IR degradation, such as oxygen vacancy migration through characterization of degraded MLCCs, is critical.

Degradation in multilayer ceramic capacitors can be characterized in several ways. Some destructive methods, such as highly accelerated lifetime testing, monitor the leakage current of the part when it is exposed to high temperature and high voltage, to assess when the current exceeds a threshold value [9, 10, 11, 12]. This method is useful for determining lifetime statistics, but lacks spatial information on the origin of failure. To understand this, imaging methods such as Infra‐Red Optical Beam Induced Resistance Change (IR‐OBIRCH) [13, 14], Voltage Contrast‐Scanning Electron Microscopy (VC‐SEM) [7, 15], and Cathodoluminescence (CL) [16] have been used to look at degraded MLCC microstructures. However, a critical drawback of such imaging techniques is that destructive cross‐sectioning is required, which limits applicability to inspection of parts lifted from mission‐critical applications. The current gold standard for evaluation of defect chemistry during exposure to electric field and temperature for dielectrics is thermally stimulated depolarization current measurements (TSDC), which is valuable for understanding the defects controlling degradation, such as oxygen vacancy space charge migration, that decrease IR [17, 18, 19, 20, 21, 22]. TSDC has been used to understand mobile charged species in dielectric materials such as Mn‐doped PZT [18], Fe‐doped SrTiO_3_ [19], BaTiO_3_ single crystals [20], and BaTiO_3_‐based ceramics used for MLCCs [17, 21, 22, 23, 24]. However, the nondestructive nature of TSDC is highly dependent on the polarization time, temperature, and electric field applied to the material; exceeding some limits can induce dielectric breakdown or trap electromigrated charged defects into an irreversible state [7, 8, 13, 14, 25]. Additionally, TSDC measures a bulk‐averaged depolarization current response of the mobilized charged defects as they relax from their metastable state, so destructive imaging methods are still required to obtain spatial information.

Nondestructive approaches for degradation and internal damage assessment of MLCCs resulting from *in operando* conditions include X‐ray [26], electrical resonance [27], acoustic resonance [28, 29, 30], and acoustic microscopy methods [31]. These methods are sensitive to various defect types in MLCCs such as internal cracks and dielectric breakdown damage. X‐ray computed tomography (XCT) is sensitive to dielectric breakdown damage [26], but it is limited by the cost of the technique and the scale of the damage relative to the imaging conditions. Electrical resonance [27], and acoustic resonance [28, 29, 30] techniques are sensitive to variations in the respective resonance behavior when large‐scale damage, i.e., cracks or delaminations, occurs within the MLCC, but these techniques lack spatial information. Acoustic microscopy methods have focused on diffraction‐limited imaging of delaminations and voids perpendicular to the wave displacement and the internal Ni electrodes [31] which lack sensitivity to damage below the diffraction limit, such as degradation induced by space charge migration. In contrast, acoustic parameters such as wave speed and attenuation can be useful for detecting defects in dielectrics at a much smaller scale [32]. Wave speed is sensitive to changes in the elastic properties of a material, e.g., those due to porosity or structural changes [33]. Attenuation is sensitive to scattering and absorbing from regions smaller than the wavelength that differ in acoustic impedance [32, 34]. Thus, the attenuation of ultrasonic waves in materials is influenced by the presence of defects. Point defects, such as vacancies and interstitial atoms, disrupt the periodicity of the crystal lattice, leading to localized variations in mass and elasticity [35]. These irregularities absorb and scatter acoustic waves, resulting in energy dissipation and increased attenuation. Thus, variations in the acoustic impedance induced by degradation can affect the attenuation in a ceramic. The extent of this absorption and scattering is frequency‐dependent; higher frequencies are more susceptible to attenuation due to the increased interaction with point defects [35]. Therefore, it is essential to utilize high‐frequency ultrasound (>50 MHz) to maximize sensitivity to microstructural degradation in MLCCs influenced by point defects such as space charge migration. This work utilizes 100 MHz center frequency ultrasonic waves to assess the thickness‐averaged attenuation response in commercial MLCCs before and after thermal and electrical degradation via TSDC.

To demonstrate the viability of high‐frequency ultrasonic attenuation for detecting microstructural degradation induced by defect electromigration in MLCCs, samples were tested ultrasonically in a water immersion system using longitudinal bulk waves, for which particle displacement is in the direction of wave propagation. Ultrasonic results are compared to passive voltage contrast (PVC) micrographs via scanning electron microscopy (SEM) of the electrically degraded samples. Structural degradation is also confirmed via HRTEM and EELS analysis (Figures S1 and S2). It is demonstrated that attenuation is sensitive to structural changes within the dielectric caused by the electromigration of oxygen vacancies, thus developing an alternative approach to identifying localized regions where severe degradation has occurred.

## 2 Evidence for Oxygen Vacancy Migration

**FIGURE 1:**
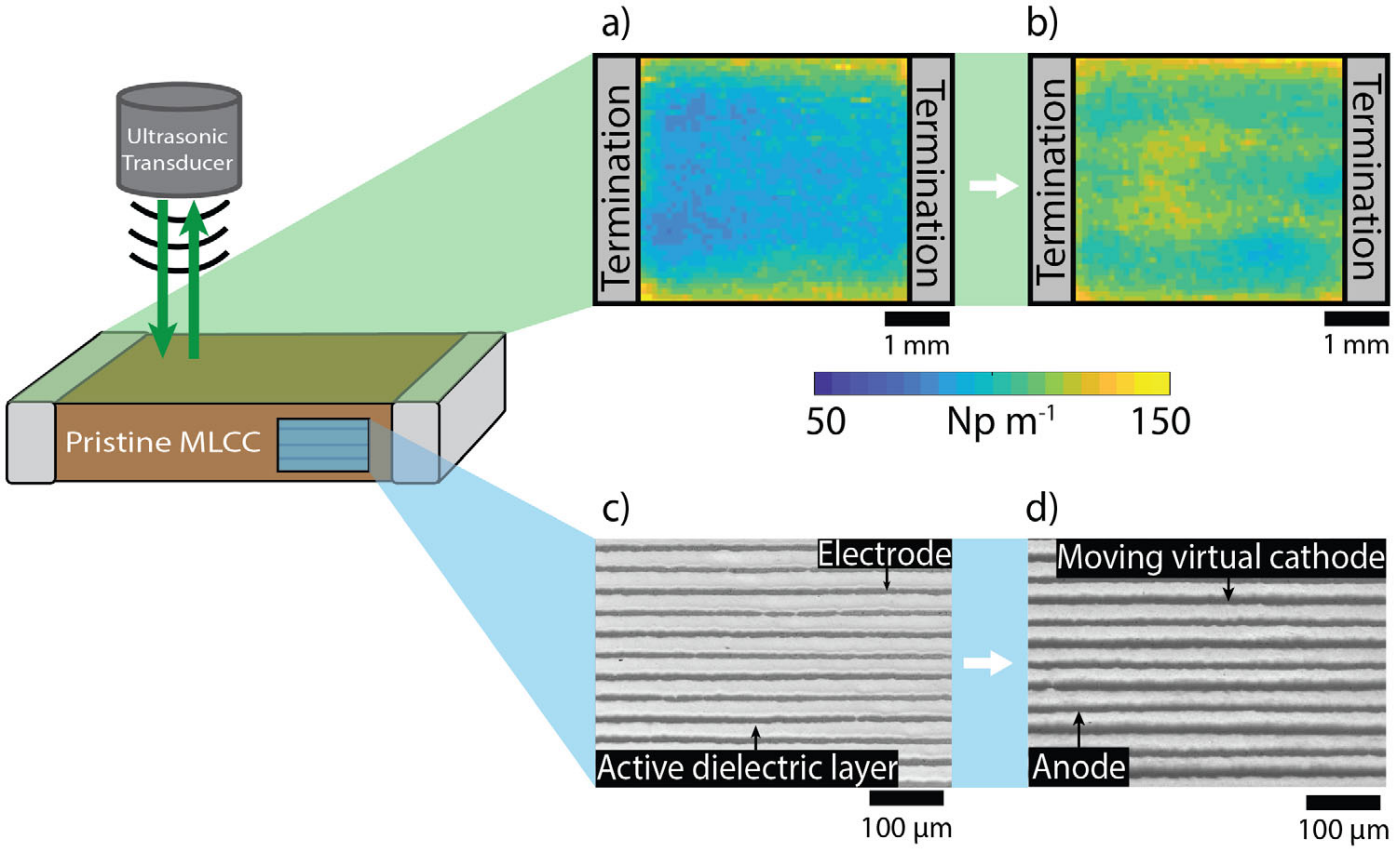
An attenuation map of (a) a pristine‐state MLCC showing homogeneous acoustic attenuation across the MLCC body and (b) an MLCC after being degraded with TSDC at 140°C and 300 V, showing more inhomogeneity across the MLCC body. SEM (c) of the cross‐sectioned (a) pristine‐state MLCC shows visually regular thicknesses of internal Ni electrodes (dark gray) and active dielectric layers (light), and SEM (d) of the cross‐sectioned (b) degraded‐state MLCC shows apparently thicker high conductivity regions near every other internal Ni electrode (dark gray), caused by oxygen vacancy migration.

Ultrasonic attenuation and wave speed measurements were performed on all MLCCs in the pristine state, and cross‐sectional PVC SEM was performed on a subset of parts to assess the local conductivity of the dielectric and the electodes. Figure 1a demonstrates an ultrasonic attenuation map that corresponds to a pristine state MLCC. The homogeneity of the ultrasonic attenuation map suggests that this part lacked significant defects that would increase ultrasonic absorption or scattering, which is expected of a pristine MLCC. Figure 1c demonstrates the cross‐sectional PVC SEM obtained from a separate pristine part, which shows expected contrast differences between the BaTiO_3_‐based dielectric layer (light gray) and the Ni electrodes (dark gray) due to differences in material conductivity [15, 36]. As expected, based on the tape casting and screen printing methods used to fabricate MLCC, each dielectric layer in the pristine part appears to be the same thickness, as does each nickel electrode layer.

**FIGURE 2:**
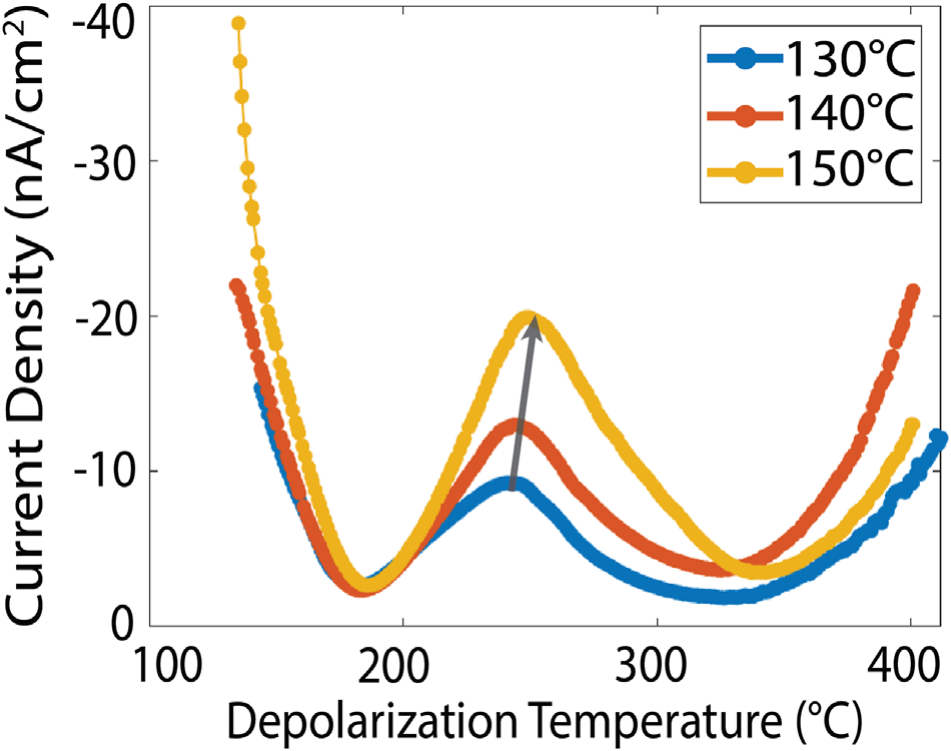
TSDC curves showing the evolution of current density as a function of temperature for three polarization temperatures (130°C, 140°C, and 150°C). The current density maxima shift toward higher temperatures with increasing polarization temperature (gray arrow), indicating space charge migration is present in these MLCCs.

Subsequently, MLCCs were degraded by TSDC, and the population of mobile charged defects was characterized through analysis of the measured depolarization currents. The depolarization current densities for MLCCs poled at 130°C, 140°C, and 150°C are plotted in Figure 2. Strong peaks are present at 229°C, 231°C, or 241°C for MLCCs poled at 130°C, 140°C, or 150°C, respectively. With increasing polarization temperature, the maximum temperature of the current density peaks shifts to higher depolarization temperatures, which is indicative of space charge migration [17, 18, 22]. In BaTiO_3_‐based dielectrics, space charge develops due to the migration of oxygen vacancies across the grains and grain boundaries toward the cathode [17, 22, 23]. Thus, TSDC of the MLCCs confirmed the presence of oxygen vacancy migration within the degraded MLCCs.

Ultrasonic attenuation and passive voltage contrast SEM (PVC SEM) were performed on the degraded MLCCs after TSDC. Figure 1b demonstrates the impact of oxygen vacancy migration on the measured ultrasonic response. The attenuation map of the degraded MLCC shows a significant increase in inhomogeneity from that of the same sample's pristine state seen in Figure 1a. Therefore, structural changes within the MLCC increased heterogeneity of the acoustic impedance within the part, thus increasing ultrasonic absorption and scattering. If the high oxygen vacancy concentration locally reduces the barium titanate composition to Ba(Ti_1/3_^4+^, Ti_2/3_^3+^)O_2.67_, then vacancy ordering (e.g. $V_{o}^{\cdot \cdot }$ ordering on every third {111} plane resulting in a 6 x d{111} supercell) can lead to a distorted perovskite as described by Woodward et al. [7]. This crystal structure change at the Ni cathode and dielectric interface would modify the acoustic impedance mismatch and thus the absorption and scattering behavior of ultrasonic waves. Moreover, it would not fully relax on depolarization. The presence of oxygen vacancy migration is further supported by the difference in the dielectric layer conductivity across the cross‐sectional PVC SEM micrograph in Figure 1d. The PVC SEM reveals the emergence of floating virtual cathodes, represented as thicker regions of increased conductivity (dark) every other electrode, due to the electromigration of oxygen vacancies under a DC field that accumulate at the cathode side and change the conductivity of the dielectric to a more semiconducting state (Figure S3).

**FIGURE 3:**
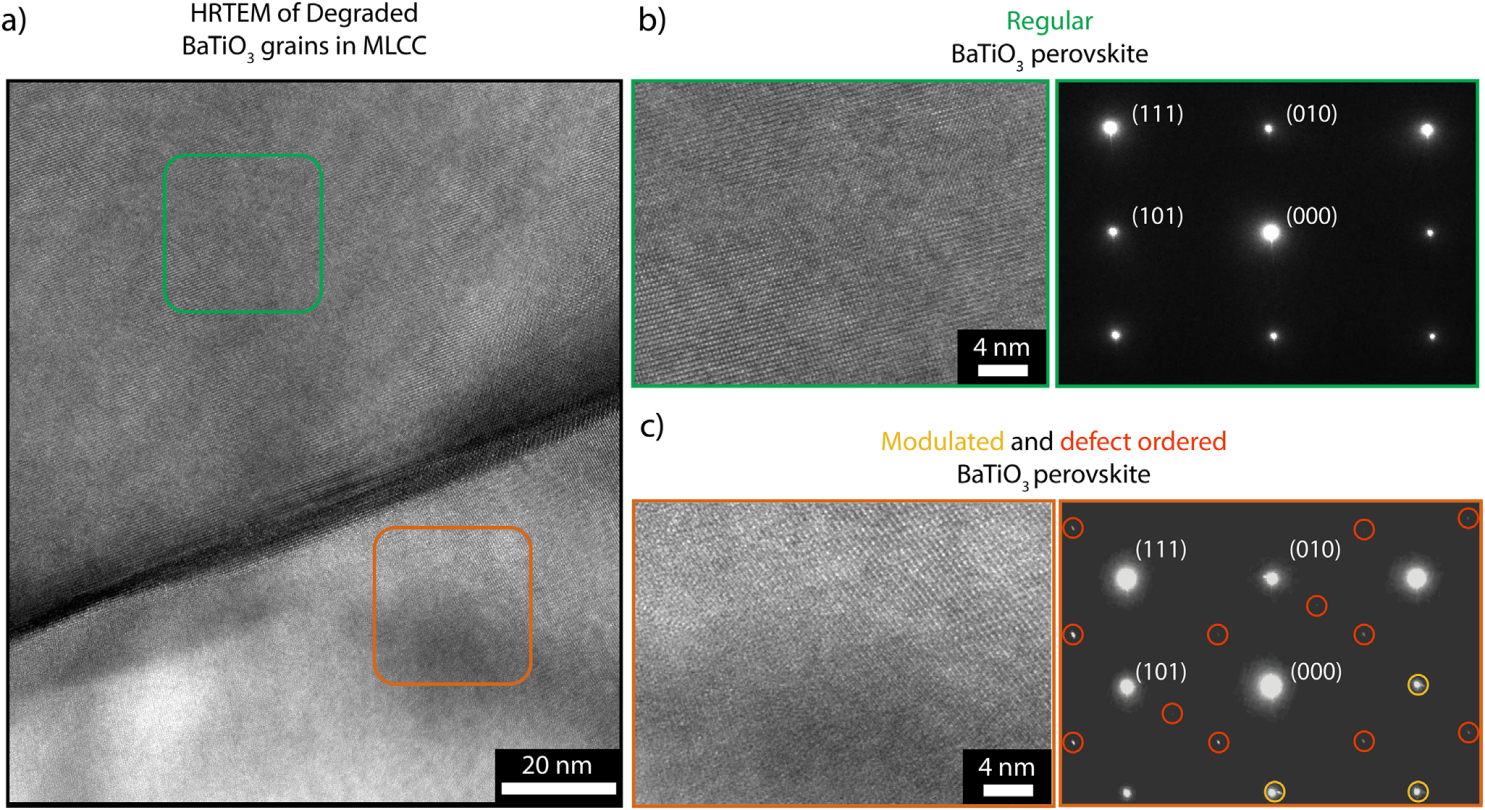
(a) High‐resolution transmission electron microscopy (HRTEM) image of a grain boundary in the MLCC. The sample is oriented such that the electric field was applied approximately vertically up during the TSDC. Thus, there should be a high concentration of oxygen vacancies near the grain boundary in the lower grain, and a much lower concentration of oxygen vacancies near the grain boundary in the upper grain. (b) Magnified region of HRTEM (green box) and Selected Area Electron Diffraction (SAED) of the regular perovskite structure showing typical perovskite symmetry. (c) Magnified region of HRTEM (orange box) and SAED of the modulated (yellow circles) and defect‐ordered (red circles) BaTiO_3_ perovskite structure showing satellite peaks and superlattice diffraction peaks.

To verify that this structural change was occurring in the degraded MLCCs, high‐resolution transmission electron microscopy was performed and selected area electron diffraction (SAED) measurements were taken on grains within the degraded dielectric near the cathode region. Recall that grain boundaries in BaTiO_3_ MLCC are partially blocking the migration of oxygen vacancies, but the oxygen vacancy motion within a grain is more facile [6]. Thus, during the TSDC measurement, oxygen vacancies that have migrated within a grain can typically relax back to a disordered configuration, except near grain boundaries or electrodes where the local oxygen vacancy rose to a point where oxygen vacancy ordering occurred. Thus, it would be expected that the ordered phase should be observed on one side of a grain boundary, but not the other. Figure 3a shows a representative grain boundary, imaged by HRTEM along the $\left[10\bar{1}\right]$ zone axis. Evidence of an ordered defect structure was not visible by HRTEM alone, in contrast to previous studies [7, 15], so SAED was conducted across the grain boundary as shown in Figure 3b,c. It is apparent that Figure 3b shows normal BaTiO_3_ (green box in Figure 3a) along the $\left[10\bar{1}\right]$ axis with the typical pseudocubic perovskite symmetry [32]. In contrast, Figure 3c (orange box in Figure 3a) exhibits a defective perovskite structure with two kinds of defects: modulation in the structure causing satellite peaks (yellow circles) and defect ordering resulting in superlattice reflections (red circles). Yang et al. [15] demonstrated these defect types in degraded BaTiO_3_‐based MLCCs and determined that the modulation and defect ordering are caused by clustering of a high concentration of oxygen vacancies in the perovskite lattice, which results in local lattice distortions. Thus, it is likely that the ultrasonic response observed in Figure 1 was influenced by the presence of these defective structures throughout the MLCC.

## 3 Ultrasonic Sensitivity to Oxygen Vacancy Induced Structural Degradation

**FIGURE 4:**
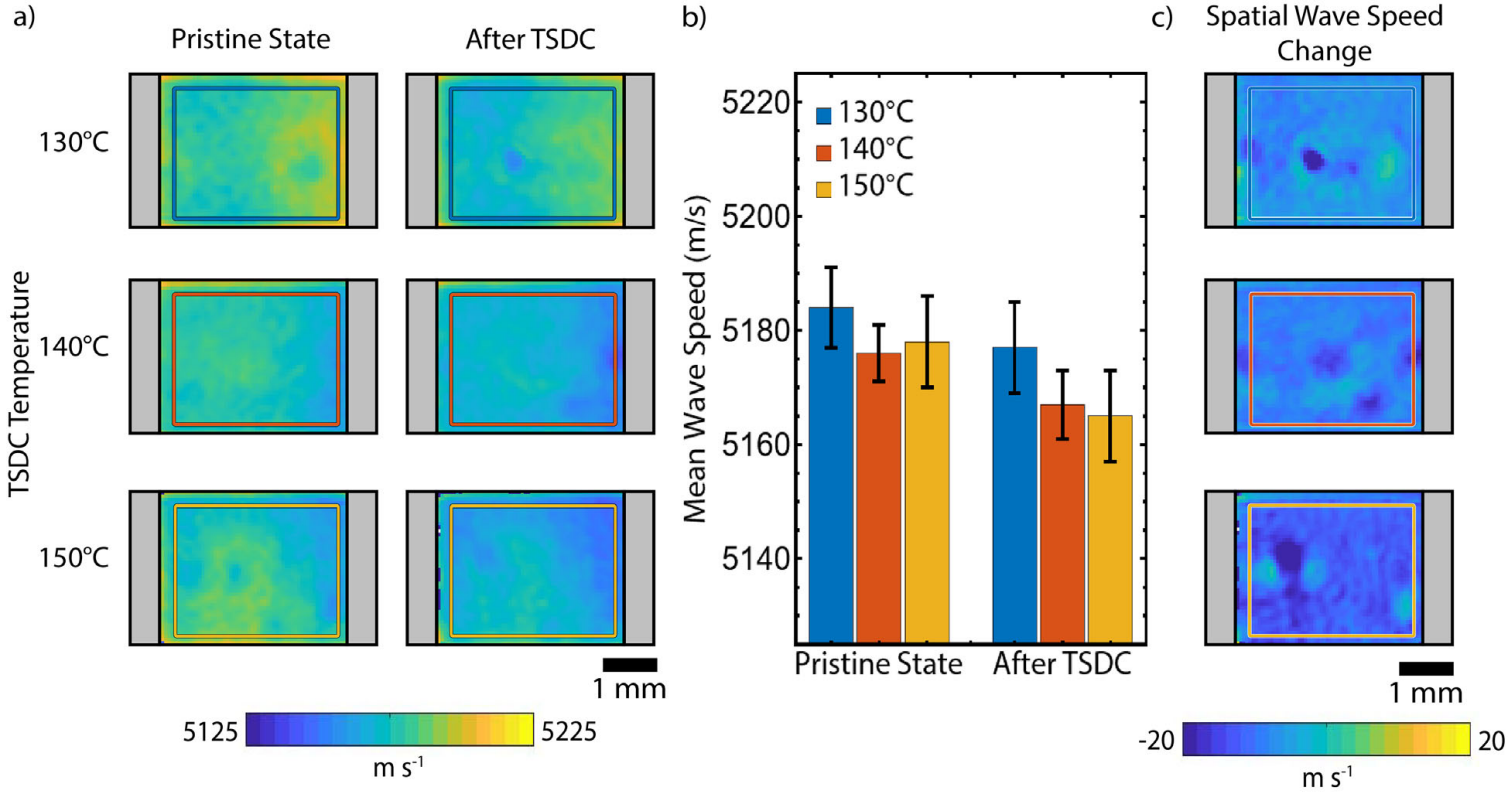
(a) Wave speed maps of the pristine state MLCCs and MLCCs after TSDC for each of the polarization temperatures (130°C, 140°C, and 150°C). (b) Bar graph of the calculated average wave speed and standard deviation from the boxed region in (a) for the pristine state MLCCs and MLCCs after TSDC. Slight decreases in the average wave speed after TSDC are consistent with internal changes to the material stiffness. Stiff ferroelectric domains, such as the *a*‐domains in BaTiO_3_, presumably contribute more to the wave speed response in pristine MLCCs, but *c*‐domains that orient along the applied electric field direction in TSDC are less stiff, which should decrease the average wave speed [38]. (c) Wave speed difference maps (maps before TSDC subtracted from maps after TSDC) showing small changes in wave speed and spatial variations for samples regardless of polarization temperature.

To further explore the sensitivity of high‐frequency ultrasound to degradation in MLCCs, the ultrasonic wave speed and attenuation were mapped before and after TSDC at the three polarization temperatures (130°C, 140°C, and 150°C). Figure 4a demonstrates ultrasonic wave speed maps corresponding to MLCCs in their pristine state and after degradation. The wave speed was averaged across a defined section within the body of the MLCC displayed as a box on the wave speed map. The mean MLCC wave speeds for all three polarization conditions show statistically insignificant decreases after TSDC, represented in the bar chart in Figure 4b, where the error bars represent the standard deviation from the mean. Slight local wave speed changes suggest changes to the material stiffness after TSDC resulting from the spontaneous polarization within ferroelectric domains, which affects the material stiffness through the soft mode [37, 38, 39]. Thus, domain orientation is a dominant mechanism dictating the material's effective stiffness. It would be expected that any residual electric field from the TSDC would preferentially favor the less stiff *c*‐domains compared to the more random domain population (including more of the stiffer *a*‐domains) in formulated BaTiO_3,_ which would decrease the average wave speed [38]. Figure 4c shows spatial difference maps for wave speed (maps before TSDC subtracted from maps after TSDC) showing spatial variations for samples for different polarization temperatures. Minor spatial variations are present; the magnitude of wave speed variation before and after TSDC (1‐3%) was inconclusive with respect to degradation within the MLCC. Thus, ultrasonic attenuation was investigated as absorption and scattering of ultrasonic waves indicate more selective sensitivity to local changes in acoustic impedance.

**FIGURE 5:**
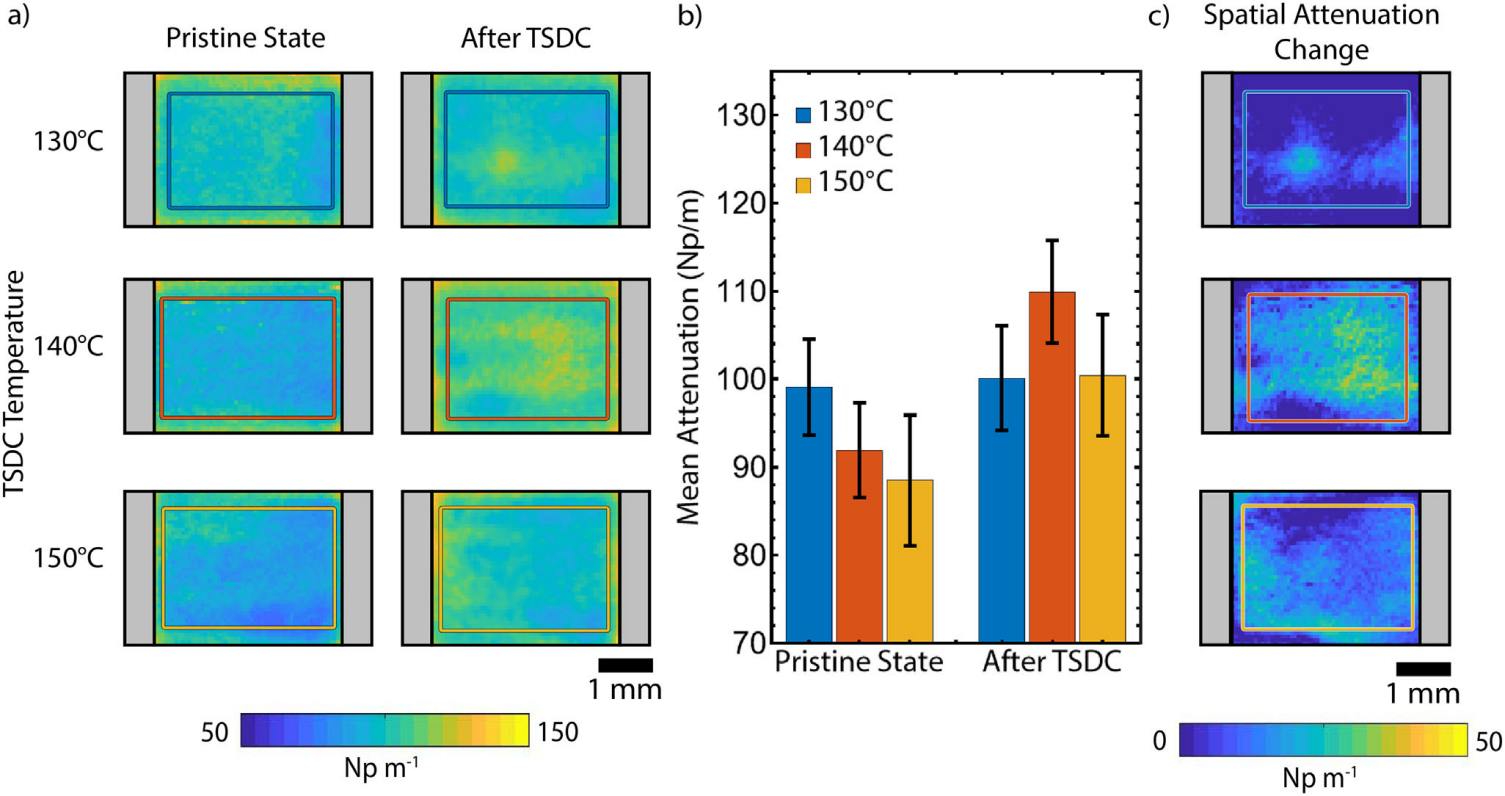
(a) Attenuation maps of the pristine state MLCCs and MLCCs after TSDC for each of the polarization temperatures (130°C, 140°C, and 150°C) showing the spatial variations caused by TSDC degradation throughout the MLCC active area. (b) Bar graph of the calculated average attenuation and standard deviation from the boxed region in (a) for the pristine state MLCCs and MLCCs after TSDC. Statistically significant increases in average attenuation after TSDC degradation are seen, implicating internal changes to the ultrasonic scattering behavior. (c) Attenuation difference maps (maps before TSDC subtracted from maps after TSDC) showing spatial variations for samples regardless of polarization temperature or statistically significant change in attenuation.

Figure 5a demonstrates ultrasonic attenuation maps corresponding to MLCCs in their pristine state and after degradation. The attenuation was averaged across a defined section within the body of the MLCC displayed as a box on the attenuation map. The mean attenuation for the lowest polarization condition (130°C) increases from 99 ± 5 Np/m to 100 ± 5 Np/m; there is no change within the standard deviation. The mean attenuation for the 140°C condition increases from 92 ± 5 Np/m to 110 ± 6 Np/m, which is 16.4% higher than the pristine state. The mean attenuation for the highest polarization condition (150°C) increases from 88 ± 5 Np/m to 100 ± 7 Np/m, 12% above the pristine state. These mean value changes are represented in the bar chart in Figure 5b, where the error bars represent the standard deviation of the mean. The attenuation changes observed for the more aggressive polarization conditions (140°C and 150°C) indicate changes occurring in the material's acoustic scattering after TSDC. This is likely due to the development of the modulated and defective structures along the cathode regions within the MLCCs, resulting in significant acoustic impedance changes when averaged through the thickness of the sample.

Moreover, high‐frequency ultrasonic attenuation measurements also introduce spatial sensitivity to the detection of oxygen vacancy‐induced degradation in MLCCs. Figure 5c shows attenuation difference maps calculated from the difference between the sample before and after TSDC and showcases spatial variations in the attenuation response after TSDC for all samples regardless of polarization condition. TSDC data can point to a complex interplay between each form of polarization (defect dipoles, the in‐grain space charge, and the transgranular space charge peaks). These are collectively related to the evolution of an internal field that opposes the driving applied field, which is statistically distributed over the sample. Thus, it is challenging to directly correlate the TSDC polarization temperature to the attenuation response. Nonetheless, there is unambiguous qualitative correlation. These data indicate that high‐frequency ultrasonic attenuation is sensitive to the acoustic impedance differences induced by structural distortions facilitated through mobilization and clustering of oxygen vacancies in BaTiO_3_‐based MLCCs; thus, it can serve as a non‐electrical detection method of this mechanism of MLCC degradation.

## 4 Conclusion

This study demonstrates the sensitivity of nondestructive ultrasonic attenuation to oxygen vacancy migration‐induced structural changes after thermally stimulated depolarization current measurements. Thus, this technique enables spatial detection of oxygen‐vacancy‐induced degradation across a 2D MLCC area utilizing through‐thickness averaged ultrasonic attenuation response. MLCCs were degraded under three different TSDC polarization conditions (130°C, 140°C, or 150°C). The maximum in the depolarization current peak recorded from TSDC shifted higher in temperature with increasing polarization condition, indicating space charge migration in the MLCCs. PVC‐SEM was performed on cross‐sectioned pristine and degraded MLCCs to confirm the presence of oxygen vacancy migration. Variations in greyscale/electrical conductivity across the internal electrode layers on the degraded MLCC cross‐section corroborated the TSDC results, indicating oxygen vacancy migration. SAED on defective BaTiO_3_ grains revealed the presence of modulated and defect‐ordered perovskite structures caused by the clustering of oxygen vacancies at individual grain boundaries. This structural change led to measurable increases in the ultrasonic attenuation response for the MLCCs tested at higher TSDC polarization conditions (140°C and 150°C). Spatial variations in the attenuation response were also recorded regardless of TSDC conditions, showing an increase in the measured attenuation within the defined area from 0.6 Np/m to 18 Np/m. This suggests spatial sensitivity to the structural changes induced by oxygen vacancy migration in BaTiO_3_‐based MLCCs, thus highlighting nondestructive ultrasonic attenuation as an alternative approach to electrical methods of identifying localized regions where severe degradation has occurred.

## 5 Experimental Section/Methods

### 5.1 Sample Preparation

Commercial 1812×7R MLCCs (100 V rated, 560 nF, 0.9% average tanδ) with Ni internal electrodes were purchased. Thicknesses for each capacitor were measured at 10 locations and determined to be approximately 1.5 mm after averaging.

### 5.2 Thermally Stimulated Depolarization Current (TSDC)

The conditions for TSDC follow those outlined in Akkopru‐Akgun et al. [15]. MLCCs were oriented vertically and pressed between electrical contact pins within a custom cube furnace. Samples were heated to the polarization temperature (130°C, 140°C, and 150°C), then electrically degraded under DC voltage (300 V) to induce electromigration of charged defects. Samples were degraded for 8 h, then cooled to room temperature, which freezes the charged defects in the degraded state. Samples were then shorted, the voltage was removed, and the samples were subsequently heated at a rate of 10°C/min from 50°C to 425°C. The relaxation current from the depolarization of the electromigrated charged defects was recorded using a B2985b electrometer (Keysight Technologies) across the temperature range. Figure 2 represents the depolarization current density measured due to the relaxation of the mobilized charged defects.

### 5.3 Ultrasonic Testing

**FIGURE 6:**
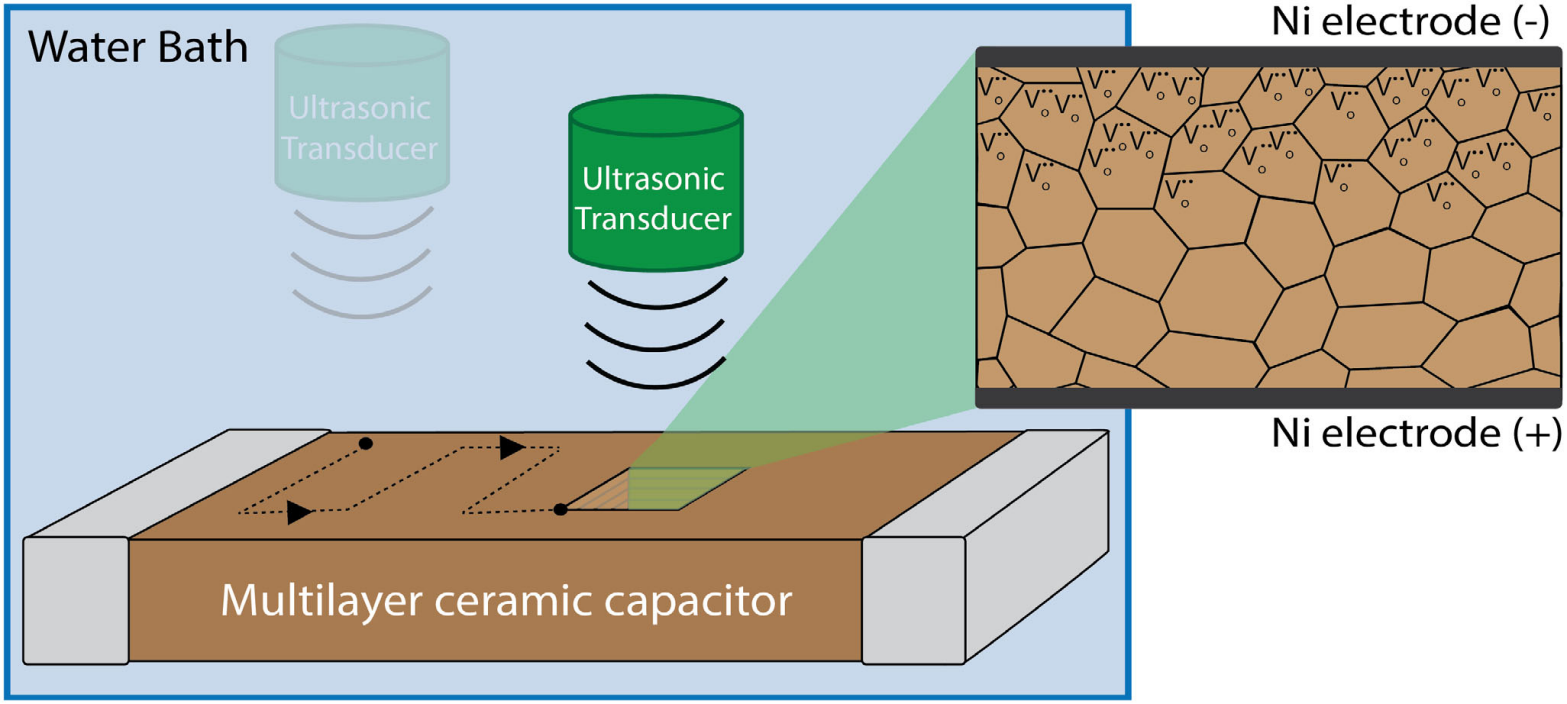
Schematic of the ultrasonic characterization setup for nondestructive testing of MLCCs. Samples are placed into a water bath, and the transducer is placed at the calculated WP to focus on the back surface of the sample. The transducer is then rastered across the sample at the designated step size, collecting through‐thickness ultrasonic information which can be related to oxygen vacancy migration.

The experimental configuration, shown in Figure 6, consisted of a pulse‐echo immersion ultrasonic setup (Mistras Group, Princeton Junction, NJ) with a single focused broadband transducer with a nominal center frequency of 100 MHz placed perpendicular to the sample surface (normal incidence). The beam width of the transducer used in this work is 0.04 mm, which serves as the diameter for the circular spot size area of 0.0013 mm^2^. The volume interrogated is the thickness multiplied by the spot size, which will vary slightly across all samples due to thickness variations. The samples were immersed in a water bath, the transducer was normalized to the top MLCC surface, and the MLCC was leveled using a manual leveling plate to ensure minimal shifts of the front and back surface reflection times of arrival (less than 0.02 µs). The surface was scanned over with a lateral step size of 0.05 mm. The probe has a focal length and element diameter of 8 and 3 mm, respectively. The water path (WP) was calculated in accordance with

(2) $$\mathrm{WP}\;=\;F\;-\;\mathrm{MP}\left(\frac{c_{m}}{c_{w}}\;\right)$$

where MP is the material path, which was selected such that the focal point location was at the approximate bottom plane of each sample (i.e., MP = sample thickness), c_m_ is the average wave speed of BaTiO_3_ (∼5300 m/s), and c_w_ is the wave speed of water (1486 m/s) [33, 40]. Due to scattering from the internal nickel electrodes, a frequency of 70 MHz produced the highest intensity signal and was used for data analysis. Time‐dependent amplitude data (Figure S4) were collected at each scanning point with a 1000 MHz sampling rate. Fifty ensemble averages were performed at each scanning point to remove Gaussian noise prior to recording the averaged waveform. Relative longitudinal attenuation (α) calculations were performed using the ratio of a silica front surface (Γ_*s*_) and the sample's back surface (Γ_*bw*_) spectral amplitudes to correct for system effects in subsequent measurements in accordance with [33]

(3) $$\alpha =-\frac{\log\left(\frac{\Gamma_{bw}}{\Gamma_{s}}\right)}{8.686\cdot MP}$$

### 5.4 Other Characterization

Capacitance and dielectric loss were measured using an LCR meter at 1 kHz and 1 V (Keysight Technologies E4980A). Samples were cross‐sectioned and mechanically polished down to 1 µm particle size (Leica TXP) to prepare for imaging using Al_2_O_3_ polishing paper. Passive voltage contrast SEM was performed using the Scios 2 scanning electron microscope. High‐resolution TEM and selected area diffraction (Talos F200×2) were performed on samples prepared by focused ion beam (FIB, Scios 2).

## Author Contributions

**Haley N. Jones**: conceptualization, methodology, data curation, visualization, writing – original draft, writing – review and editing, validation. **Andrea P. Argüelles**: conceptualization, validation, supervision, writing – review and editing, funding acquisition. **Susan Trolier‐McKinstry**: conceptualization, validation, supervision, writing – review and editing, funding acquisition. **Clive A. Randall**: writing – review and editing, supervision, validation.

## Funding

The authors acknowledge support from The Center for Dielectrics and Piezoelectrics, under the auspices of the Industry/University Cooperative Research Centers program of the National Science Foundation via Grant Nos. IIP‐1841453 and IIP‐1841466. This material is based upon work supported by the National Science Foundation Graduate Research Fellowship Program under Grant No. DGE1255832. Any opinions, findings, and conclusions or recommendations expressed in this material are those of the author(s) and do not necessarily reflect the views of the National Science Foundation.

## Conflicts of Interest

The authors declare no conflicts of interest.

## Supporting information




**Supporting File 1**: advs77083‐sup‐0001‐SuppMat.docx.

**Supporting File 2**: advs77083‐sup‐0002‐FigureS1–S4.zip.

## Data Availability

The data that support the findings of this study are available from the corresponding author upon reasonable request.
