# Supplementary material for: Detection of Degraded Formulated BaTiO3 Induced by Oxygen Vacancy Migration Using Ultrasonic Attenuation
Source: Adv Sci (Weinh). 2026 Aug 14:e77083. Online ahead of print. doi: 10.1002/advs.77083 (PMC13474181; doi:10.1002/advs.77083)
Supplement: Supplementary file 1 [file ADVS-9999-e77083-s001.docx]

Supporting Information

Detection of Degraded Formulated BaTiO_3_ Induced by Oxygen Vacancy Migration Using Ultrasonic Attenuation

*Haley N. Jones, Clive A. Randall, Andrea P. Argüelles*, Susan Trolier-McKinstry**

**Supplementary Figure S1**. (a) Low magnification TEM image of a pristine MLCC showing the formulated-BaTiO_3_ microstructure and a low density of core-shell grains and (b) higher magnification TEM of the boxed grains in (a).

**Supplementary Figure S2**. Electron Energy Loss Spectroscopy (EELS) showing the Ti L_2,3_ (top) and O K (bottom) edges of barium titanate in a pristine MLCC (blue) and a TSDC degraded MLCC (orange). The Ti L_2,3_ lacks a shift to lower energy loss in the degraded state which could indicate that Ti present in the pristine MLCC was already in a reduced state. The increased width of the Ti L_2,3_ peaks from 9.8 ± 0.1 eV to 10.1 ± 0.1 eV indicates further Ti reduction after degradation. The O K edge contains four peaks labeled A-D. The decrease in peak A after degradation implies a disruption of the linear Ti-O bonds while maintaining octahedral coordination of the oxygen atoms which results in strain relaxation. Reduction of peaks C and D after degradation is associated with a higher concentration of oxygen vacancies.^[7]^

**Supplementary Figure S3**. (a) Standard SEM of a degraded MLCC cross-section and (b) PVC-SEM of a degraded MLCC cross-section highlighting differences in conductivity at the electrode and dielectric layers. In (b), the Ni electrodes appear darker than the dielectric layer (light gray) due to higher conductivity. Extended dark regions away from the electrode in (b) that alternate every other electrode showcases a floating virtual cathode caused by oxygen deficient degraded BaTiO_3_-based dielectric which is more metallic than non-degraded BaTiO_3_.^[7]^

**Supplementary Figure S4**. (a) A-scan of the pristine state MLCC and (b) A-scan of the same MLCC after degradation via TSDC. The reflections corresponding to the front and back surfaces of the MLCC are labeled. Variations are seen in the front and back surface reflections, but it is difficult to interpret thus necessitating calculation of ultrasonic attenuation.
